# Supplementary material for: Exploring Impacts of a Nutrition-Focused Massive Open Online Course
Source: Nutrients. 2022 Sep 6;14(18):3680. doi: 10.3390/nu14183680 (PMC9500789; doi:10.3390/nu14183680)
Supplement: Supplementary file 1 [file nutrients-14-03680-s001.zip › Supplementary Table S1 Food as Medicine MOOC outline.pdf]

Supplementary Table S1. Food as Medicine MOOC outline

Week	Theme	Topic	Delivery Mode
1. Food as Medicine	Introduction	Welcome to the course	Article
		Course philosophy	Article
		Safe communication of nutrition information	Article
		Is it 'food' or is it 'medicine'	Video
	History of Food as Medicine	Food as medicine in history	Video
		How and where have foods been used as medicine in the past?	Article
		How food has been used as medicine	Video
		The importance of evidence for food as medicine	Article
	Food in Health and Disease	The role food can play in prevention and treatment	Article
		Try three new foods	Article
		Foods and prevention	Video
		Foods and treatment	Video
		Nutrition and the immune system	Video
		Can the use of food as medicine go too far?	Article
		How many different foods do you have everyday?	Article
	What's in food that makes it special?	The food matrix	Video
		Macronutrients	Video
		Micronutrients	Video
		Phytochemicals and phytonutrients	Video
		Superfoods and phytochemicals	Quiz
		Superfoods: myth or real?	Article
	Foods and Inflammation	What is inflammation and how is it useful?	Article
		Acute local inflammation and wound healing	Article
		Chronic inflammation and rheumatoid arthritis	Article

			Foods and inflammation	Video
		Weekly Feedback	Recap the Week	Video
			Take the first steps in joining a global network or nutrition educators	Article
2.	A Body Systems Approach for Food as Medicine	Food and the Gut	Welcome to Week 2	Article
			Fibre, prebiotics and the gut	Article
			The importance of dietary fibre for health	Article
			Food and fibre	Article
			What is the difference between prebiotics and probiotics?	Article
			Diet and gut symptoms – The Low FODMAP Diet for IBS	Article
		Food and the Brain	What is appetite and how can it affect my health?	Article
			What controls my appetite?	Video
			Food and appetite	Article
			Is food quality important?	Video
			Food addiction	Article
		Food and our Genome	What are genes and why are they important for health?	Video
			The Human Genome Project	Video
			What our can our genes tell us?	Video
			Food, Nutrition and our Genes	Video
			Genes and nutrition	Quiz
		Food, Fertility and Pregnancy	Are food and nutrition important for fertility?	Article
			Which foods are important for pregnancy?	Video
			Do women need to eat for two in pregnancy?	Article
			Pre-pregnancy diet	Quiz

	Food and Weight	When is someone considered overweight?	Article
		What factors affect a person's weight?	Article
		Weight regain: why does this happen so often?	Video
		Is dieting the answer?	Video
	Weekly Feedback	Recap the week	Video
3. Interpreting the Science of Food as Medicine	Introduction	Welcome to week 3	Article
		Popular diets	Article
	Nutrition Complexities and Controversies	Factors influencing what we eat	Video
		The evolution of nutrition science	Video
		How nutrition misinformation may end up as fact	Article
		Complexities and controversies	Article
	Making Choices: Food and Diet	The challenge with portion sizes	Video
		Australian dietary guidelines	Video
		International health guidelines	Article
		Guidelines to health eating	Quiz
	Who Can You Trust?	Nutrition information: What to look out for	Video
		Popular diets - revisited	Article
	Applying Food as Medicine to You	Mindful eating: introduction	Video
		How to eat mindfully	Video
		Mindful eating: discretionary foods	Video
	Where Is the Future Heading?	The future of foods	Video
		What are functional foods?	Article
		Personalised nutrition	Article
	Additional Resources	Supporting information	Article
		Acknowledgments	Article
	Weekly Feedback	Recap the week	Article

	End of Course	Discussion
	Take the first steps in joining a global network of nutrition educators	Article
