# Supplementary material for: Exploring Impacts of a Nutrition-Focused Massive Open Online Course
Source: Nutrients. 2022 Sep 6;14(18):3680. doi: 10.3390/nu14183680 (PMC9500789; doi:10.3390/nu14183680)
Supplement: Supplementary file 1 [file nutrients-14-03680-s001.zip › Supplementary Table S2 pre course survey questions.pdf]

Supplementary Table S2: Pre-course Survey Questions and Response Options

Questions	Responses
What is your age?	<18yrs
	18-25yrs
	26-35yrs
	36-45yrs
	46-55yrs
	56-65yrs
	>65yrs
Which country are you from?	List of all countries
Have you studied nutrition before?	Yes- at university or research institute
	Yes – at college or TAFE
	Yes – at high school
	Yes- short course
	No – I haven't studied nutrition before
Please indicate the most appropriate response that best describes your level of nutrition knowledge	I have no knowledge about nutrition
	I only know a little nutrition information
	I know the basics of nutrition information
	I have a good general knowledge of nutrition
	I am very knowledgeable about nutrition
How many courses have you studied online?	0
	1
	2
	3
	4
	More than 5
Is this the first MOOC (Massive Open Online Course) you have participated in?	Yes
	No
	Unsure
What is your main reason for taking this course?	Career prospects
	Self-knowledge
	Help others
	Help with future study
	Topic interests me
	It was suggested to me
	Other – free text box
