# Supplementary material for: Exploring Impacts of a Nutrition-Focused Massive Open Online Course
Source: Nutrients. 2022 Sep 6;14(18):3680. doi: 10.3390/nu14183680 (PMC9500789; doi:10.3390/nu14183680)
Supplement: Supplementary file 1 [file nutrients-14-03680-s001.zip › Supplementary Table S3 post course survey questions.pdf]

Supplementary Table S3: Post-course Survey Questions and Response Options

| Questions | Responses |
| --- | --- |
| How satisfied were you with the course? | Extremely satisfied |
| | Somewhat satisfied |
| | Neither satisfied no dissatisfied |
| | Somewhat dissatisfied |
| | Extremely dissatisfied |
| How relevant was the content of the course for you and your goals? | Very relevant |
| | Somewhat relevant |
| | Neither relevant nor irrelevant |
| | Somewhat irrelevant |
| | Very irrelevant |
| Did you find the comment in the discussion forums from other learners useful? | Very useful |
| | Somewhat useful |
| | Not very useful |
| | Definitely not useful |
| | I did not read any comments in the discussion forums from other learners |
| How likely are you to apply what you have learnt? | Extremely likely |
| | Somewhat likely |
| | Neither likely nor unlikely |
| | Somewhat unlikely |
| | Extremely unlikely |
| How confident are you in applying what you have learnt? | Extremely confident |
| | Somewhat confident |
| | Not very confident |
| | Not confident at all |
| How likely are you to continue seeking further information on nutrition? | Extremely likely |
| | Somewhat likely |
| | Neither likely nor unlikely |
| | Somewhat unlikely |
| | Extremely unlikely |
