# Supplementary material for: Exploring Impacts of a Nutrition-Focused Massive Open Online Course
Source: Nutrients. 2022 Sep 6;14(18):3680. doi: 10.3390/nu14183680 (PMC9500789; doi:10.3390/nu14183680)
Supplement: Supplementary file 1 [file nutrients-14-03680-s001.zip › Supplementary Table S4 Fruit and Veg survey questions.pdf]

Supplementary Table S4: Fruit and Vegetable Survey Questions and Response Options

Questions	Response options
After completing this course, how many serves of vegetables do you now eat each day? (NOTE: 1 serve of vegetables = 1 cup of salad vegetables (e.g. tomato, lettuce, cucumber), OR 1/2 cup cooked vegetables	A little less (1-2 serves less)
	A lot less (2+ serves less)
	Same
	A little more (1-2 serves)
	A lot more (2+serves more)
In total, how many serves of vegetables do you usually eat each day?	0-1
	2
	3
	4
	5
	6 or more
After completing this course, how many serves of fruit do you now eat each day? (NOTE: 1 serve of fruit = 1 medium piece of fruit (e.g. an apple), OR 2 small pieces of fruit (e.g. 2 fresh apricots), OR 1 cup of fruit (e.g. strawberries or chopped fruit salad)	A little less (1-2 serves less)
	A lot less (2+ serves less)
	Same
	A little more (1-2 serves)
	A lot more (2+serves more)
In total, how many serves of fruit do you eat each day?	0-1
	2
	3
	4
	5
	6 or more
